# Dielectric and Measurement-Domain Feature Fusion for Machine Learning-Based Broadband Microwave Sensing: Application to Ethanol Concentration Estimation

**DOI:** 10.3390/s26206512

**Published:** 2026-10-14

**Authors:** Abdullah Oguz Kizilcay, Fidan Gamze Kizilcay

**Affiliations:** 1 Computer Engineering Department, Zonguldak Bülent Ecevit University, Zonguldak 67100, Türkiye; 2 Department of Information Technology, Zonguldak Bülent Ecevit University, Zonguldak 67100, Türkiye; gamze.kizilcay@beun.edu.tr

**Keywords:** ethanol concentration estimation, feature fusion, machine learning, microwave sensing

## Abstract

This study investigates the predictive capability of different microwave-derived feature types for machine learning-based ethanol concentration estimation in water–ethanol mixtures. Eleven mixtures were prepared at 10 wt.% concentration intervals, and cyclic measurements were performed over the 2–6 GHz frequency range to construct an experimental dataset. The input variables included conventional dielectric parameters together with reflection-based measurement features. Three ensemble regression models, namely Random Forest, Extra Trees, and XGBoost, were evaluated using group-wise 5-fold cross-validation, with repeated measurements grouped according to their measurement cycles. The results demonstrated highly accurate concentration prediction from microwave measurements, with the Extra Trees regressor achieving the best overall performance. Feature analysis further showed that a hybrid feature set consisting of the real permittivity ($\varepsilon^{\prime }$) and the real part of the reflection response (Refl.R) was the best-performing reduced feature set. Under group-wise 5-fold cross-validation, this compact two-feature model produced prediction performance comparable to that of the full seven-feature model, yielding a mean RMSE of 0.1135 wt.% with a standard deviation of 0.0864 wt.%. Additional leave-one-concentration-out validation further showed that the proposed feature set remained the best-performing combination for unseen intermediate concentration levels, achieving an RMSE of 2.5501 wt.% and an R^2^ of 0.9902. These findings highlight the potential of combining measurement-domain and dielectric features to develop compact and accurate prediction models for broadband microwave sensing.

## 1. Introduction

Ethanol is an important chemical widely used in the energy, agricultural, food, pharmaceutical, cosmetic, and chemical industries due to its versatility as a fuel, solvent, disinfectant, preservative, and industrial raw material [1,2,3,4]. As the commercial value and demand for ethanol-based products continue to increase, concerns related to product quality, dilution, and adulteration have also become more significant in both industrial and consumer markets [5]. In particular, fraudulent practices such as unauthorized water addition, methanol contamination, or mislabeling may lead to economic losses, reduced product performance, and serious public health risks [6,7]. For this reason, considerable research efforts have been devoted to rapid and reliable ethanol adulteration detection and concentration determination [8,9,10].

Microwave, optical, ultrasonic, and spectroscopic techniques have all been investigated for alcohol–water mixture characterization and adulteration analysis. Early studies mainly focused on direct dielectric characterization using vector network analyzers with open-ended probes [11], where the concentration-dependent behavior of the real and imaginary permittivity components was experimentally analyzed. Similar characterization-oriented approaches were also reported in ultrasonic sensing systems [12], where signal decomposition techniques such as SSA and PCA were employed to estimate mixture composition from acoustic responses. Although these studies provide valuable physical insight into mixture behavior, they are generally based on analytical interpretation or handcrafted signal descriptors rather than end-to-end predictive frameworks.

A large number of studies have focused on microwave resonator-based liquid sensors, including OCSRR sensors [13], LC and microfluidic resonators [14], cavity resonators [15,16], metamaterial-assisted sensors [17], CELC/SRR structures [18], and differential resonant platforms [19,20]. In these approaches, concentration or dielectric properties are typically inferred from resonance frequency shifts, attenuation levels, quality factors, or fitted calibration equations. While many of these systems achieve high sensitivity and low error, they generally exploit only a small subset of the measured electromagnetic response, potentially neglecting complementary information available in the raw measurement-domain data.

Machine learning (ML)-assisted sensing has also emerged as an alternative to conventional laboratory-grade instrumentation. Low-cost- and low-range-based microwave resonators [21], RFID-enabled passive sensors [22], and thermally modulated metal-oxide gas sensors [23] have demonstrated that multidimensional signal fingerprints can be directly mapped to target variables using ML models. These studies confirm that data-driven methods can significantly improve the practicality, portability, and real-time usability of low-cost sensing platforms. However, issues related to model generalization, interpretability, and long-term measurement stability are still not consistently addressed.

In parallel, optical and photonic sensing approaches have shown strong promise for alcohol authentication and adulteration detection. Fluorescence nanoparticle sensors [24], porous silicon photonic structures [25], lossy mode resonance fiber sensors [26,27], chiral photonic films [28], resonant nanopillar devices [29], and Raman spectroscopy systems [30] have all been applied to ethanol or methanol analysis. Depending on the sensing platform, ML models such as Random Forest, SVM, ANN, CNN, SVR, and Gaussian Process Regression achieved high classification or regression performance, often exceeding 97% accuracy or R^2^ values above 0.95. However, many of these methods rely on optical spectra, simulated datasets, or high-dimensional signals that require substantial preprocessing, feature engineering, or specialized sensing materials.

Considering the above-mentioned limitations of existing microwave sensing approaches, broadband dielectric spectroscopy remains the preferred experimental technique whenever high measurement accuracy and reliable dielectric characterization are required. Owing to its broadband measurement capability, open-ended coaxial probe systems provide not only the dielectric parameters extracted after calibration but also the original electromagnetic reflection response from which these parameters are derived. However, most studies use only the derived dielectric parameters, while the original reflection information is ignored. Whether this information can improve machine-learning-based sensing has not yet been investigated.

The present study investigates a more effective use of broadband dielectric spectroscopy data for machine-learning-based ethanol concentration prediction. Water–ethanol mixtures were measured using an open-ended coaxial probe, and the predictive capabilities of conventional dielectric parameters and measurement-domain features were systematically compared.

## 2. Materials and Methods

### 2.1. Materials and Sample Preparation

Water and ethanol were used as the base materials to prepare the samples. Distilled water was used throughout the experiments, while the ethanol used in this study had a stated purity of 99% and was obtained from the university laboratory. Since water and ethanol have different densities, the mixture ratios were determined on a mass basis rather than a volume basis to ensure higher accuracy in composition.

The required amounts of water and ethanol were measured using a precision balance with a resolution of 0.01 g. The tare method was applied during weighing to minimize measurement errors. Each component was carefully added to the container according to the predefined ratios.

The detailed composition of each sample is presented in Table 1.

**Figure 1:**
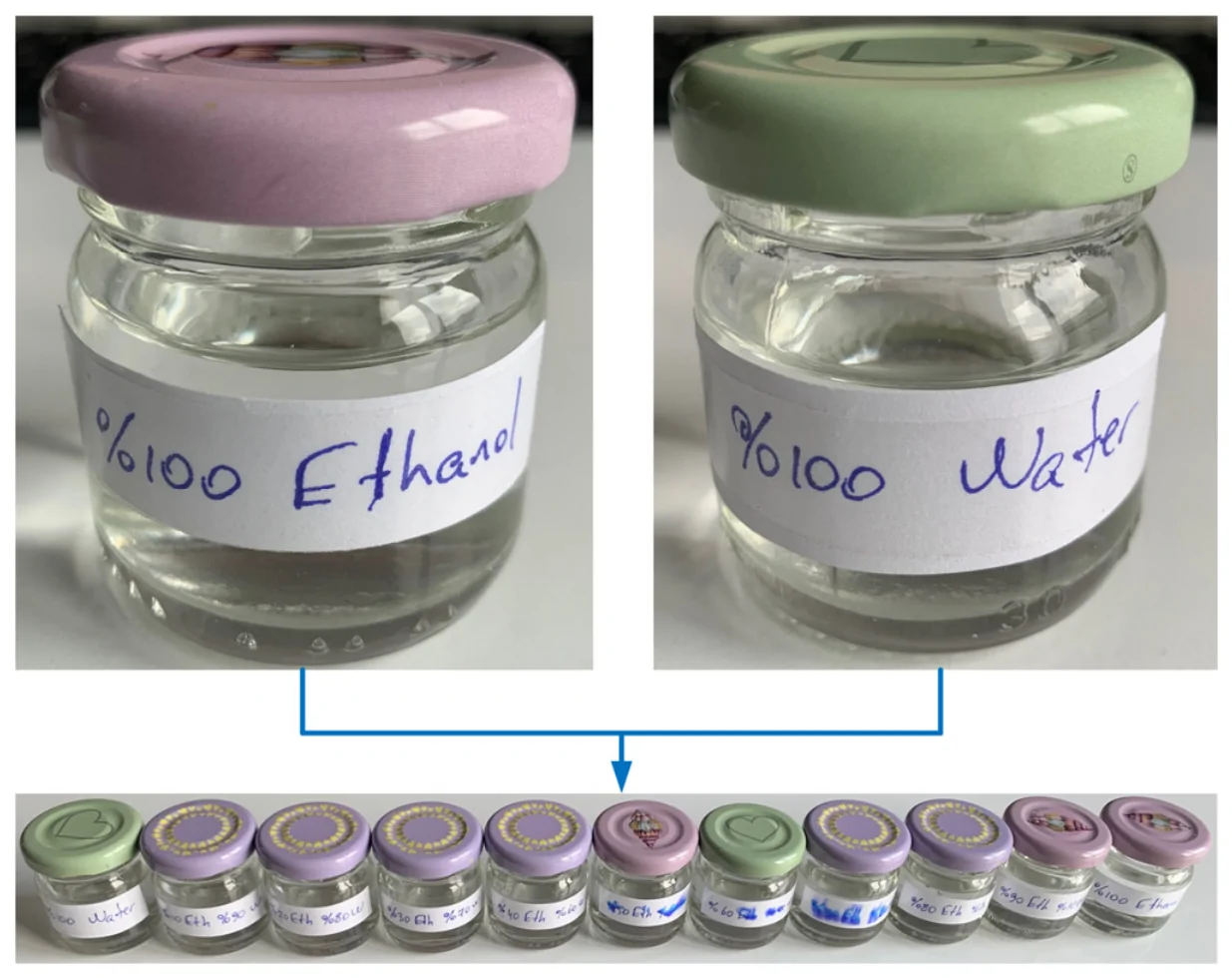
Sample preparation of water–ethanol mixtures.

Table 1 summarizes the compositions of the 11 prepared mixtures, covering ethanol concentrations from 0% to 100% in 10% increments. One physical mixture was prepared for each concentration level, resulting in a total of 11 independently prepared samples. The prepared samples are shown in Figure 1.

During sample preparation, water was added to the glass containers prior to ethanol to reduce evaporation effects. The mixtures were then gently agitated to ensure homogeneity.

### 2.2. Measurement System

Dielectric measurements were carried out using an open-ended coaxial probe system connected to a Vector Network Analyzer (VNA). In this study, a DAK3.5 Dielectric Assessment Kit (SPEAG, Zurich, Switzerland) was used together with a Rohde & Schwarz ZNL20 VNA (Rohde & Schwarz, Munich, Germany) to obtain the frequency-dependent dielectric response of the samples.

Maintaining a stable probe position and ensuring full contact between the sensing surface and the sample are critical for achieving accurate results. Therefore, a mechanical holder was used to keep the probe fixed and to minimize any movement during the measurements. The system was calibrated once prior to the measurements using a standard open–short–load (OSL) procedure.

During the measurements, the probe was immersed in the liquid sample. The electromagnetic signal generated by the VNA was transmitted into the sample through the probe, and the reflected signal was recorded in terms of S-parameters. The reflection response is physically related to the dielectric properties of the material through the electromagnetic impedance [31]. However, the measured reflection response does not directly provide the dielectric properties of the sample. In open-ended coaxial probe measurements, the complex permittivity is obtained from the measured reflection response through calibration, electromagnetic modeling, and an inversion procedure. Accordingly, reflection coefficient (Refl.R and Refl.I) represents the directly measured reflection-domain response, whereas $\varepsilon^{\prime }$ and $\varepsilon^{\prime \prime }$ represent dielectric quantities derived from this response.

In machine learning, features derived from original measurements can provide useful transformed representations of the measured data and improve predictive performance [32,33]. Based on this feature-engineering principle, both directly measured reflection-domain quantities and derived dielectric quantities were investigated as candidate features in this study. Their predictive contributions were then evaluated through different feature combinations.

**Figure 2:**
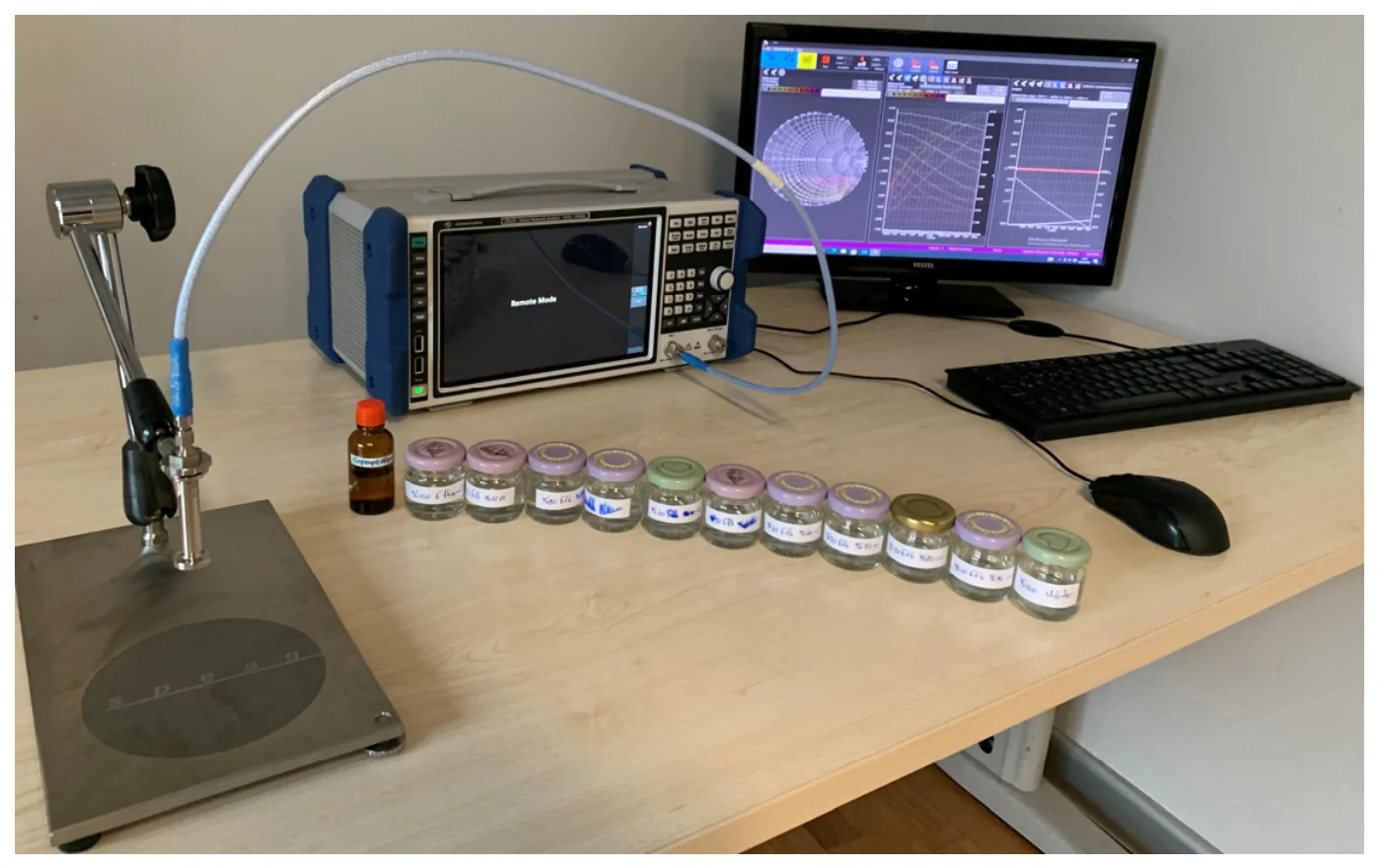
Experimental setup for measuring the reflection response of liquid samples using a VNA and an open-ended coaxial probe.

All measurements were conducted at 25 °C. The measurement setup is illustrated in Figure 2.

The measurements were conducted in 10 cycles. In each cycle, all 11 samples were measured sequentially from Sample 1 to Sample 11. This cyclic procedure was used instead of performing consecutive repetitions of the same sample to reduce potential systematic and time-dependent effects. Each measurement was performed over the 2–6 GHz range using a 50 MHz frequency step, yielding 81 frequency points per measurement. After each measurement, the container was tightly closed, and the probe tip was cleaned with isopropyl alcohol to prevent cross-contamination.

### 2.3. Machine Learning Regression Model

ML is a branch of artificial intelligence that enables computers to learn patterns from data and perform tasks such as regression, classification, and prediction without explicit rule-based programming [34,35]. In this work, three tree-based ensemble regression models were employed: Random Forest (RF), Extra Trees (ETs), and XGBoost (XGB) [36,37,38]. These methods were selected because of their robustness, ability to capture nonlinear relationships, and suitability for feature importance analysis.

The initial feature set consisted of frequency and dielectric parameters, including the real and imaginary parts of permittivity ($\varepsilon^{\prime }$ and $\varepsilon^{\prime \prime }$), electrical conductivity (σ), and loss tangent (tanδ). Reflection components representing the real and imaginary parts of the measured Refl.R and Refl.I were also included. Measurements were performed over the 2–6 GHz frequency range, which provides stable dielectric measurements while covering the frequency region where the dielectric response of water–ethanol mixtures exhibits clear variation. A frequency step size of 50 MHz was used, yielding 81 frequency points per mixture. Each of the 11 water–ethanol mixtures was cyclically measured 10 times to improve reliability and reduce measurement variability. Consequently, the final dataset comprised 8910 observations, each represented by 7 input features.

The computational analysis was implemented in Python 3.10.19 on Windows 10 using scikit-learn 1.7.2 and XGBoost 3.2.0. No normalization or standardization was applied to the input features, and the features were used in their original numerical scales.

The RF model used 300 estimators with max_features = 1.0 and bootstrap sampling, while the ET model used 300 estimators with max_features = 1.0 and bootstrap sampling disabled. The XGB model used 500 estimators, a learning rate of 0.05, a maximum depth of 6, subsample = 0.9, and colsample_bytree = 0.9. These hyperparameters were predefined and remained fixed throughout the analyses.

To obtain a robust evaluation, model performance was assessed using Group 5-Fold Cross-Validation instead of a conventional random train–test split. Repeated measurements were grouped according to their measurement cycle, ensuring that observations from the same measurement cycle were not distributed between the training and test sets.

An additional nested group-wise cross-validation was performed to separate model and feature-set selection from performance evaluation. The outer loop used Group 5-Fold Cross-Validation for performance evaluation, while model and feature-set selection was performed exclusively within the corresponding outer-training data using Group 4-Fold Cross-Validation. In each inner loop, all predefined feature sets and the three regression models were evaluated, and the best-performing combination was selected. Model hyperparameters were predefined and remained fixed throughout the cross-validation procedure.

In addition to the group-wise validation procedures, a leave-one-concentration-out (LOCO) validation was performed to evaluate generalization to unseen concentration levels. In each iteration, all observations corresponding to one ethanol concentration were completely excluded from model training and used only for testing. For the assessment of interpolation performance, the intermediate concentration levels from 10 to 90 wt.% were considered, whereas the boundary concentrations were excluded because their removal requires extrapolation beyond the concentration range represented in the training data. For each excluded concentration, the test set comprised 810 observations, corresponding to 81 frequency points across 10 measurement cycles. Predictions were generated individually for all observations without averaging across frequency points or measurement cycles. For the overall LOCO evaluation, the predictions from the nine intermediate concentrations were pooled, resulting in 7290 predictions.

To identify the most informative features, multiple feature combinations were evaluated. Model performance was assessed using Root Mean Squared Error (RMSE), Mean Absolute Error (MAE), and the coefficient of determination (R^2^), defined as [39]

(1) $$RMSE=\sqrt{\frac{1}{n}\sum_{i=1}^{n}{\left(y_{i}-y_{i}^{\prime }\right)}^{2}}$$

(2) $$MAE=\frac{1}{n}\sum_{i=1}^{n}\left|y_{i}-y_{i}^{\prime }\right|$$

(3) $$R^{2}=1-\frac{\sum_{i=1}^{n}{\left(y_{i}-y_{i}^{\prime }\right)}^{2}}{\sum_{i=1}^{n}{\left(y_{i}-\bar{y}\right)}^{2}}$$

where *n* is the number of test samples, $y_{i}$ and $y_{i}^{\prime }$ denote the true and predicted values, respectively, and $\overset{ˉ}{y}$ represents the mean of the observed values. RMSE and MAE quantify the magnitude of prediction errors, while R^2^ indicates the proportion of variance explained by the model. RMSE was used as the criterion for model and feature-set selection.

Feature importance analysis was performed using the selected regression model to quantify the relative contribution of each input feature [36] to ethanol concentration prediction. In addition, Spearman rank correlation analysis was conducted to evaluate the monotonic relationship between each input feature and ethanol concentration.

### 2.4. Feature Trend Analysis

In order to interpret the contribution of the selected features, it is essential to analyze their behavior over the frequency range of interest. Examining their variation provides insight into their relevance and consistency with respect to ethanol concentration. To enable a direct comparison of the trends of considered features, all variables were normalized using min–max normalization, as given by [40,41].

(4) $$X_{norm}=\frac{X-X_{min}}{X_{max}-X_{min}}$$

This approach scales each feature to the range [0, 1], eliminating the effect of differing magnitudes and units. Since the objective is to analyze the variation patterns of the features with respect to ethanol concentration rather than their absolute values, normalization provides a clearer representation of their relative behavior.

## 3. Results

### 3.1. Dielectric Measurement Results

**Figure 3:**
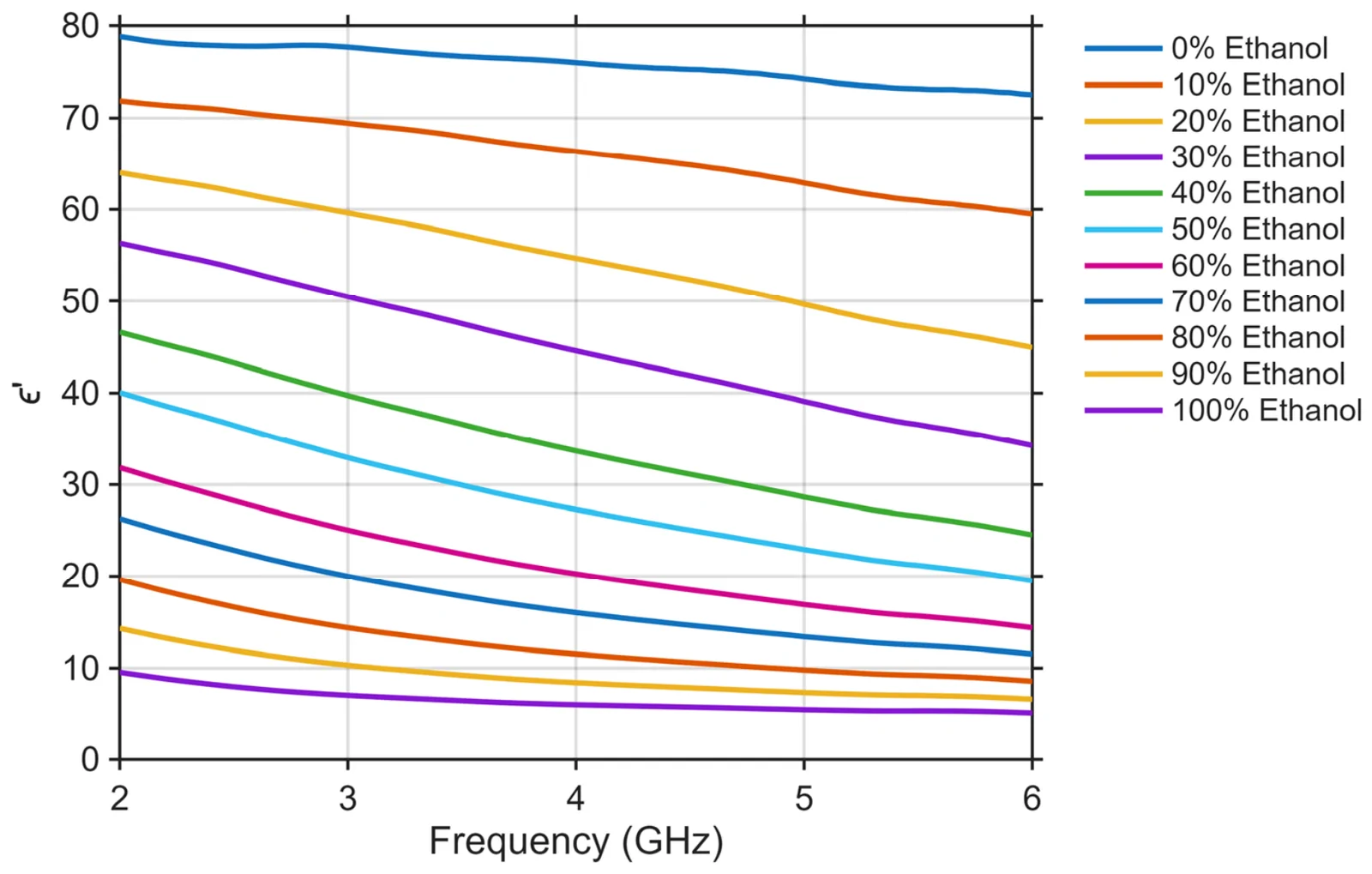
Mean $\varepsilon^{\prime }$ of the samples for different ethanol concentrations.

The dielectric properties of the samples were measured 10 times to evaluate measurement consistency. The repeated measurements showed close agreement with only minor variations, indicating good measurement repeatability under the investigated conditions. Figure 3 presents the mean dielectric constant ($\varepsilon^{\prime }$) calculated from the 10 repeated measurements for each ethanol concentration.

As shown in Figure 3, the averaged dielectric constant decreases monotonically with both increasing frequency and ethanol concentration. The curves corresponding to different ethanol concentrations are clearly separated over the entire frequency range, indicating strong discriminative characteristics in the measured dielectric response.

To quantitatively evaluate measurement repeatability, the standard deviation was calculated from the 10 repeated measurements at each concentration and frequency point. The mean standard deviation across all concentration–frequency combinations was 0.292 for $\varepsilon^{\prime }$, corresponding to a mean coefficient of variation of 0.9%. For Refl.R, the mean standard deviation was 0.00477. These results indicate low variability among the repeated measurements.

### 3.2. Model Performance Comparison

To provide a robust performance assessment, all regression models were evaluated using group-wise 5-fold cross-validation. Therefore, the reported metrics represent the mean and standard deviation across the five folds.

The comparative performance of the evaluated models is summarized in Table 2.

As shown in Table 2, the ET regressor achieved the best overall performance, with a mean RMSE of 0.1262, a mean MAE of 0.0298, and a mean R^2^ of 0.999977. It also yielded the lowest standard deviations for all evaluation metrics. Although XGB and RF also achieved high prediction accuracy, both models produced higher prediction errors and larger standard deviations than ET.

### 3.3. Correlation Analysis and Feature Importance

To better understand the contribution of each variable to ethanol concentration prediction and to identify the most informative features, Spearman correlation coefficients and feature importance values obtained from the ET model trained using all seven input features are summarized in Table 3. Such analyses are particularly useful for larger and more complex datasets, where reducing redundant features can improve computational efficiency and model interpretability.

As shown in Table 3, $\varepsilon^{\prime }$ exhibited the strongest correlation with ethanol concentration and was identified as the most influential feature by the ET model. Refl.R showed the second-highest correlation and feature importance, indicating that it also plays a significant role in the prediction process. Although tanδ and Refl.I also showed relatively strong correlations with ethanol concentration, their contributions to the prediction model remained noticeably lower than those of $\varepsilon^{\prime }$ and Refl.R. In contrast, conductivity and frequency contributed only marginally to the prediction performance.

**Figure 4:**
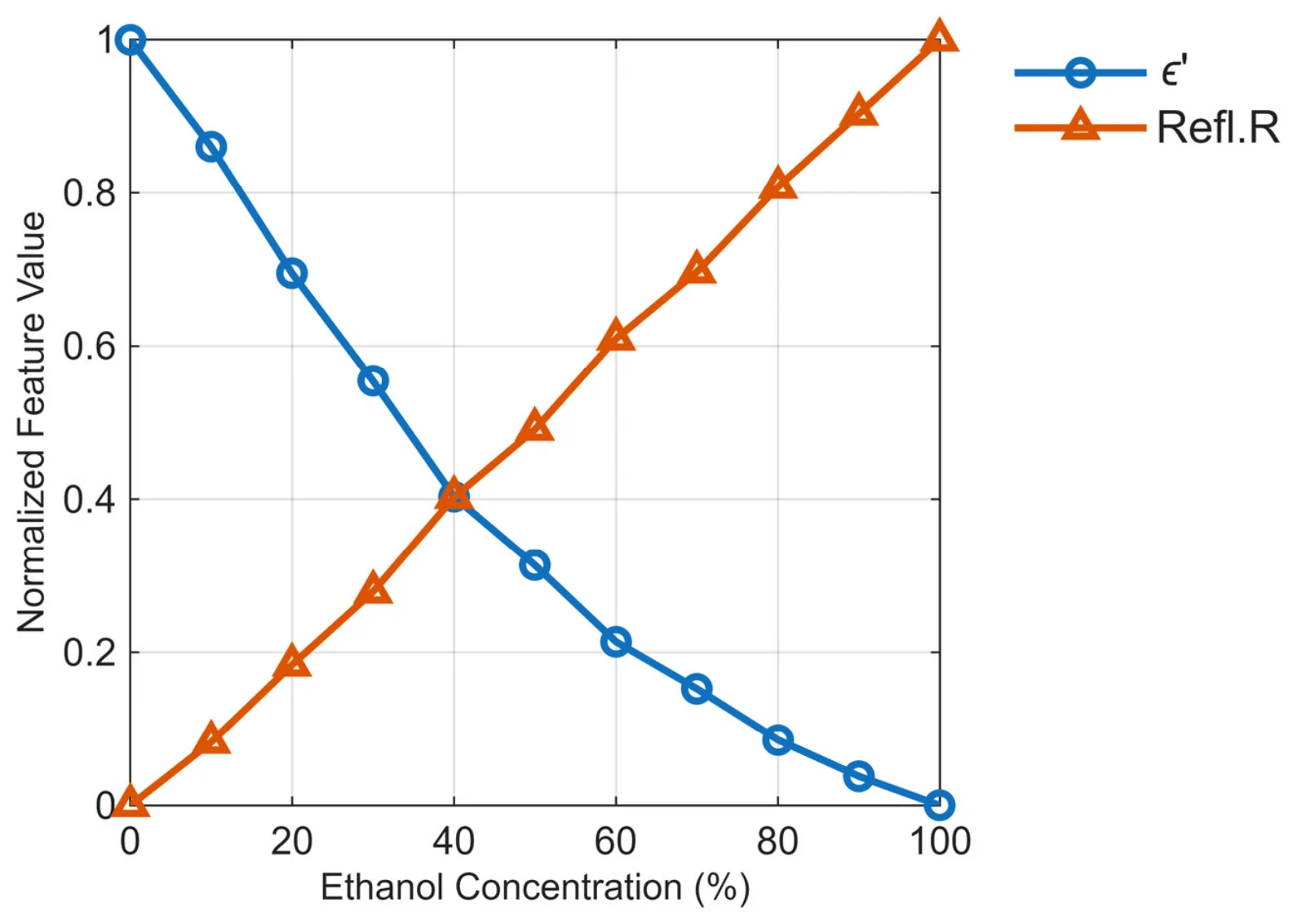
Normalized trends of the two most informative features.

A notable observation is that $\varepsilon^{\prime }$ and Refl.R showed strong correlations of similar magnitude but opposite direction with ethanol concentration. While $\varepsilon^{\prime }$ decreased as ethanol concentration increased, Refl.R exhibited the opposite trend and ranked second in feature importance. As shown in Figure 4, $\varepsilon^{\prime }$ decreases almost linearly with increasing ethanol concentration, whereas Refl.R exhibits a nearly symmetric increasing trend.

Although $\varepsilon^{\prime }$ is derived from the measured complex reflection response and is therefore physically related to Refl.R, the two features do not exhibit identical concentration-dependent trends. The inversion process transforms the measured reflection response into a dielectric-domain representation, while Refl.R retains the directly measured reflection-domain representation. From a machine-learning perspective, these different representations may provide useful feature diversity, which can help explain the strong predictive performance observed when $\varepsilon^{\prime }$ and Refl.R are used together.

### 3.4. Feature-Set Performance and Validation

Based on the correlation analysis and feature importance ranking, representative feature sets were selected for further evaluation. The most influential individual feature, $\varepsilon^{\prime }$, was adopted as the baseline feature set. The conventional dielectric-only feature set consisted of $\varepsilon^{\prime }$ and $\varepsilon^{\prime \prime }$, which together represent the complex permittivity commonly used for dielectric characterization. Similarly, Refl.R and Refl.I, representing the original measurement-domain information, were selected as the measurement-only feature set. The final feature set was the proposed hybrid feature set, constructed by combining $\varepsilon^{\prime }$ with Refl.R, the most informative dielectric and measurement-domain features, respectively. The corresponding results are presented in Table 4.

As shown in Table 4, prediction performance improved substantially when more informative feature combinations were used. The baseline $\varepsilon^{\prime }$ feature set produced an RMSE of 8.0778, while the dielectric-only and measurement-only feature sets reduced the RMSE to 0.9334 and 0.4768, respectively. The proposed $\varepsilon^{\prime }$ + Refl.R feature set achieved an RMSE of 0.1135, comparable to the 0.1262 obtained using all seven features, while requiring only two input features. This result supports the selection of $\varepsilon^{\prime }$ + Refl.R as a compact feature set with comparable predictive performance to the full feature set.

The model and feature-set selection was further evaluated using the nested group-wise cross-validation. The ET regressor with the $\varepsilon^{\prime }$ + Refl.R feature set was selected in all five outer folds. The resulting outer-fold performance was an RMSE of 0.1135 ± 0.0865 wt.%, MAE of 0.0248 ± 0.0193 wt.%, and R^2^ of 0.99998 ± 0.00002.

As an additional benchmark, the selected $\varepsilon^{\prime }$ + Refl.R feature set was evaluated using conventional Linear Regression and Polynomial Regression under the same Group 5-Fold Cross-Validation procedure. Linear Regression yielded a mean RMSE of 4.4778, while second-, third-, and fourth-degree Polynomial Regression yielded mean RMSE values of 1.2136, 0.7280, and 0.7284, respectively. Increasing the polynomial degree from three to four provided no further improvement.

To further evaluate the generalization of the selected feature sets to unseen concentration levels, an additional LOCO validation was performed. The Extra Trees results for the intermediate concentrations from 10 to 90 wt.% are presented in Table 5.

As shown in Table 5, the proposed $\varepsilon^{\prime }$ + Refl.R feature set achieved the best performance, with an RMSE of 2.5501 wt.%, MAE of 2.1863 wt.%, and R^2^ of 0.9902.

To further examine the LOCO performance of the selected $\varepsilon^{\prime }$ + Refl.R feature set, the results were evaluated separately for each excluded concentration, as presented in Table 6.

As shown in Table 6, the mean predicted concentrations remained close to the corresponding excluded concentration levels and followed the increasing concentration trend.

**Figure 5:**
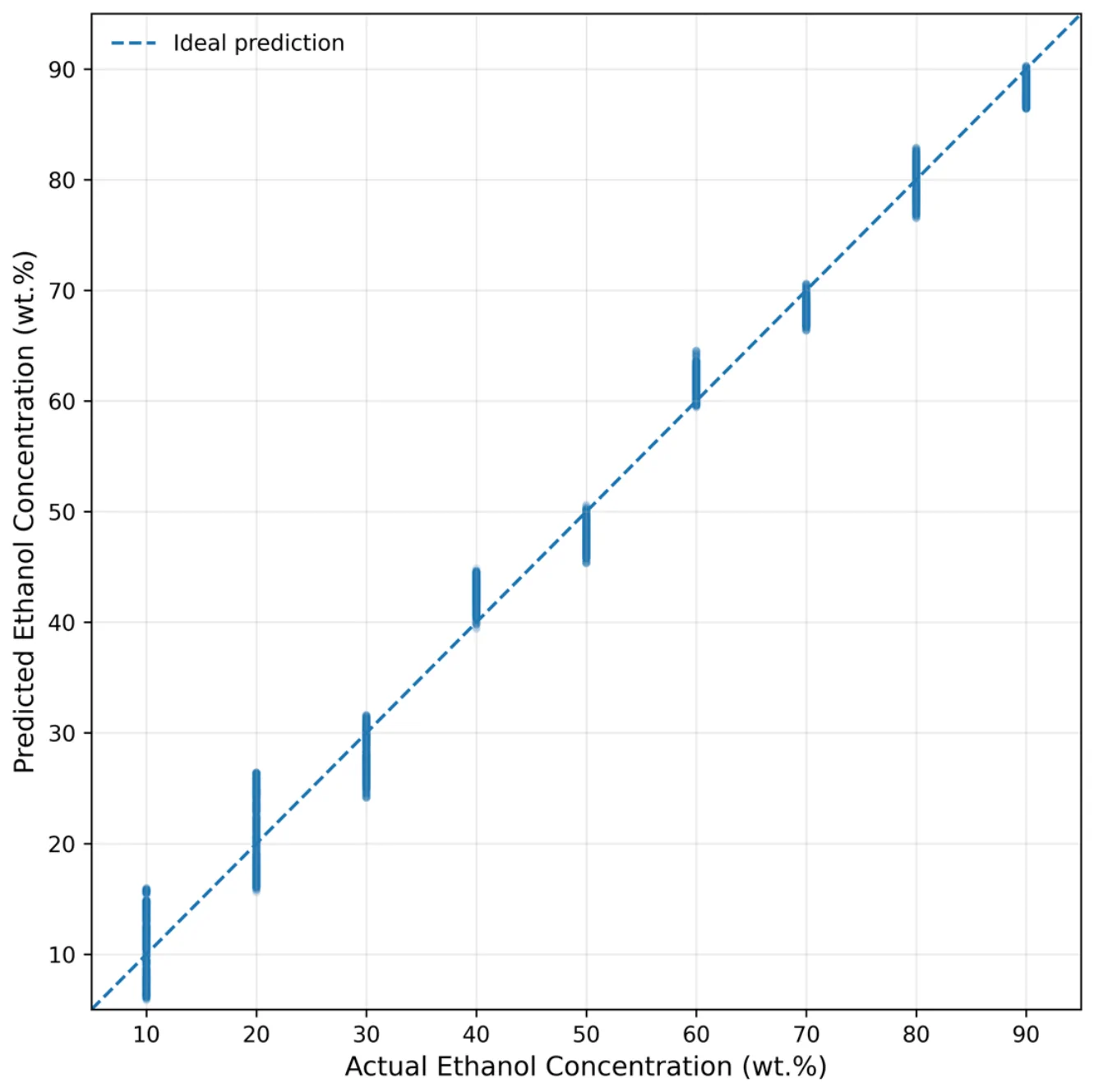
Predicted versus actual ethanol concentrations under LOCO validation.

The predicted-versus-actual concentrations obtained with this feature set are shown in Figure 5.

As shown in Figure 5, the predictions closely follow the ideal prediction line across the unseen intermediate concentration levels, while some variation is observed around individual concentrations. This visual agreement is consistent with the LOCO results.

## 4. Discussion

The repeated measurements showed low variability in both $\varepsilon^{\prime }$ and Refl.R, indicating good repeatability of the experimental measurements under the investigated conditions. The cyclic measurement procedure also allowed measurement variability to be assessed across separate measurement cycles. However, repeated measurements of the same physical preparation do not capture preparation-to-preparation variability. Future studies could therefore include multiple independently prepared samples at each concentration level to assess this source of variability.

In the initial model comparison, the ET regressor yielded the lowest prediction errors among the three evaluated ensemble models. Its performance was also supported by the conventional regression benchmark, where linear and polynomial regression produced higher prediction errors using the same $\varepsilon^{\prime }$ + Refl.R feature set. Using the selected $\varepsilon^{\prime }$ + Refl.R feature set, ET subsequently achieved a very low RMSE of 0.1135 wt.% under Group 5-Fold Cross-Validation. However, the group-wise cross-validation primarily evaluates prediction consistency across separate measurement cycles. Since different cycles contain repeated measurements of the same physical preparations, this low RMSE should be interpreted in this context rather than as prediction performance for independently prepared samples. The low error is also consistent with the low measurement variability observed in the repeatability analysis. To complement this evaluation, LOCO was additionally performed by completely excluding one concentration level from training. The RMSE increased from 0.1135 to 2.5501 wt.%, reflecting the more demanding evaluation setting in which LOCO requires prediction of concentration levels not represented in the training data. Nevertheless, the mean predictions followed the increasing ethanol concentrations, with concentration-specific RMSE values ranging from 1.4556 to 3.6166 wt.%, indicating that the overall concentration-dependent trend was preserved.

Feature analysis identified $\varepsilon^{\prime }$ and Refl.R as the two most informative descriptors. This selection was further supported by the nested validation, where the ET regressor with $\varepsilon^{\prime }$ + Refl.R was selected in all five outer folds. The same feature combination also remained the best-performing feature set under LOCO validation.

The individual roles of these two features were further examined through their concentration-dependent behavior. Although $\varepsilon^{\prime }$ and Refl.R exhibited opposite trends with increasing ethanol concentration, both varied systematically with mixture composition. Based on these observations, $\varepsilon^{\prime }$ was first selected as the baseline feature. Representative dielectric-only, measurement-only, and hybrid feature sets, together with the full seven-feature set, were then evaluated to assess the contribution of each feature category.

The proposed $\varepsilon^{\prime }$ + Refl.R feature set outperformed the other reduced feature sets and provided comparable predictive performance to the full seven-feature model while requiring only two input features. The effectiveness of the feature combination can be related to the different roles of $\varepsilon^{\prime }$ and Refl.R in the measurement-processing chain. While $\varepsilon^{\prime }$ provides the derived dielectric representation, Refl.R provides the directly measured reflection-domain representation, allowing the learning model to capture predictive patterns that are not equally represented by either feature alone.

## 5. Conclusions

This study proposes a feature fusion strategy that combines dielectric and measurement-domain features for machine learning-based broadband microwave sensing. Using ethanol concentration estimation as a representative application, the proposed $\varepsilon^{\prime }$ + Refl.R feature set outperformed the other reduced feature sets, achieving an RMSE of 0.1135 wt.%, and provided comparable predictive performance to the full seven-feature model while requiring only two input features.

Moreover, under the additional LOCO validation, the proposed feature set remained the best-performing combination for unseen intermediate concentration levels, with an RMSE of 2.5501 wt.%. The concentration-specific results further showed that the mean predictions followed the increasing ethanol concentration levels. These findings highlight the potential of feature fusion for developing compact and accurate machine learning models for broadband microwave sensing and motivate further investigation across different sensing configurations and applications.

## Figures and Tables

**Table 1:** Water–Ethanol Mixtures.

Sample No	Water Ratio		Ethanol Ratio	
	Ratio (%)	Weight (g)	Ratio (%)	Weight (g)
1	100	20	0	0
2	90	18	10	2
3	80	16	20	4
4	70	14	30	6
5	60	12	40	8
6	50	10	50	10
7	40	8	60	12
8	30	6	70	14
9	20	4	80	16
10	10	2	90	18
11	0	0	100	20

**Table 2:** 5-Fold summary of the models.

Model	Mean RMSE	Std_RMSE	Mean MAE	Std_MAE	Mean R^2^	Std_R^2^
ET	0.1262	0.0951	0.0298	0.0226	0.999977	0.000025
XGB	0.5280	0.2683	0.0815	0.0411	0.999664	0.000235
RF	0.6193	0.3400	0.0803	0.0453	0.999524	0.000377

**Table 3:** Feature importance values obtained from the model.

Feature	Spearman Correlation	Feature Importance
$\varepsilon^{\prime }$	−0.977	0.446
*Refl.R*	0.951	0.257
tanδ	0.870	0.137
*Refl.I*	0.872	0.068
$\varepsilon^{\prime \prime }$	−0.667	0.061
σ(S/m)	−0.564	0.026
*f* (MHz)	0	0.002

**Table 4:** Performance comparison of representative dielectric, measurement-domain, and hybrid feature sets using the ET regressor.

Category	Feature Set	Mean RMSE	Std_RMSE	Mean MAE	Std_MAE	Mean R^2^	Std_R^2^
Baseline	$\varepsilon^{\prime }$	8.0778	0.2502	5.5302	0.2484	0.934698	0.003990
Dielectric-only	$\varepsilon^{\prime }$+ $\varepsilon^{\prime \prime }$	0.9334	0.1612	0.1894	0.0735	0.999108	0.000295
Measurement-only	Refl.R + Refl.I	0.4768	0.1011	0.0613	0.0246	0.999764	0.000102
Hybrid (Proposed)	$\varepsilon^{\prime }$+ Refl.R	0.1135	0.0864	0.0248	0.0192	0.999981	0.000020
All seven features	Full	0.1262	0.0951	0.0298	0.0226	0.999977	0.000025

**Table 5:** ET performance under LOCO validation for unseen intermediate ethanol concentrations (10–90 wt.%).

Feature Set	RMSE (wt.%)	MAE (wt.%)	R^2^
$\varepsilon^{\prime }$	11.8881	11.0091	0.7880
$\varepsilon^{\prime }$+ $\varepsilon^{\prime \prime }$	6.8148	5.9788	0.9303
Refl.R + Refl.I	3.8641	3.1594	0.9776
All seven features	2.8867	2.4302	0.9875
$\varepsilon^{\prime }$+ Refl.R	2.5501	2.1863	0.9902

**Table 6:** Concentration-specific LOCO validation results for the ET using the $\varepsilon^{\prime }$ + Refl.R feature set.

Excluded Concentration (wt.%)	Mean Prediction (wt.%)	RMSE (wt.%)	MAE (wt.%)
10	10.9682	3.0790	2.7343
20	21.5063	3.6166	3.2439
30	28.0479	2.6385	2.1855
40	42.8109	2.9960	2.8157
50	47.9331	2.3544	2.0763
60	61.6365	1.9571	1.6650
70	68.0723	2.1002	1.9420
80	79.6922	1.4556	1.2321
90	88.2230	2.0309	1.7819

## Data Availability

Data will be made available on request.
